# Deep learning in cancer diagnosis, prognosis and treatment selection

**DOI:** 10.1186/s13073-021-00968-x

**Published:** 2021-09-27

**Authors:** Khoa A. Tran, Olga Kondrashova, Andrew Bradley, Elizabeth D. Williams, John V. Pearson, Nicola Waddell

**Affiliations:** 1 Department of Genetics and Computational Biology, QIMR Berghofer Medical Research Institute, Brisbane, 4006 Australia; 2 School of Biomedical Sciences, Faculty of Health, Queensland University of Technology (QUT), Brisbane, 4059 Australia; 3 Australian Prostate Cancer Research Centre - Queensland (APCRC-Q) and Queensland Bladder Cancer Initiative (QBCI), Brisbane, 4102 Australia; 4 Faculty of Engineering, Queensland University of Technology (QUT), Brisbane, 4000 Australia

**Keywords:** Artificial intelligence, Deep learning, Multi-modal learning, Explainability, Cancer genomics, Precision oncology, Cancer of unknown primary, Molecular subtypes, Prognosis, Tumour microenvironment, Pharmacogenomics

## Abstract

Deep learning is a subdiscipline of artificial intelligence that uses a machine learning technique called artificial neural networks to extract patterns and make predictions from large data sets. The increasing adoption of deep learning across healthcare domains together with the availability of highly characterised cancer datasets has accelerated research into the utility of deep learning in the analysis of the complex biology of cancer. While early results are promising, this is a rapidly evolving field with new knowledge emerging in both cancer biology and deep learning. In this review, we provide an overview of emerging deep learning techniques and how they are being applied to oncology. We focus on the deep learning applications for omics data types, including genomic, methylation and transcriptomic data, as well as histopathology-based genomic inference, and provide perspectives on how the different data types can be integrated to develop decision support tools. We provide specific examples of how deep learning may be applied in cancer diagnosis, prognosis and treatment management. We also assess the current limitations and challenges for the application of deep learning in precision oncology, including the lack of phenotypically rich data and the need for more explainable deep learning models. Finally, we conclude with a discussion of how current obstacles can be overcome to enable future clinical utilisation of deep learning.

## Background

Artificial intelligence (AI) encompasses multiple technologies with the common aim to computationally simulate human intelligence. Machine learning (ML) is a subgroup of AI that focuses on making predictions by identifying patterns in data using mathematical algorithms. Deep learning (DL) is a subgroup of ML that focuses on making predictions using multi-layered neural network algorithms inspired by the neurological architecture of the brain. Compared to other ML methods such as logistic regression, the neural network architecture of DL enables the models to scale exponentially with the growing quantity and dimensionality of data [1]. This makes DL particularly useful for solving complex computational problems such as large-scale image classification, natural language processing and speech recognition and translation [1].

**Fig. 1:**
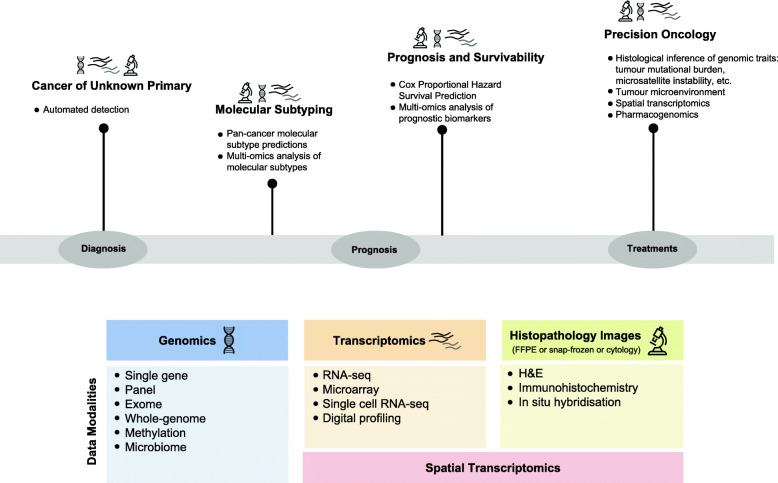
Deep learning may impact clinical oncology during diagnosis, prognosis and treatment. Specific areas of clinical oncology where deep learning is showing promise include cancer of unknown primary, molecular subtyping of cancers, prognosis and survivability and precision oncology. Examples of deep learning applications within each of these areas are listed. The data modalities utilised by deep learning models are numerous and include genomic, transcriptomic and histopathology data categories covered in this review

Cancer care is undergoing a shift towards precision healthcare enabled by the increasing availability and integration of multiple data types including genomic, transcriptomic and histopathologic data (Fig. 1). The use and interpretation of diverse and high-dimensionality data types for translational research or clinical tasks require significant time and expertise. Moreover, the integration of multiple data types is more resource-intensive than the interpretation of individual data types and needs modelling algorithms that can learn from tremendous numbers of intricate features. The use of ML algorithms to automate these tasks and aid cancer detection (identifying the presence of cancer) and diagnosis (characterising the cancer) has become increasingly prevalent [2, 3]. Excitingly, DL models have the potential to harness this complexity to provide meaningful insights and identify relevant granular features from multiple data types [4, 5]. In this review, we describe the latest applications of deep learning in cancer diagnosis, prognosis and treatment selection. We focus on DL applications for omics and histopathological data, as well as the integration of multiple data types. We provide a brief introduction to emerging DL methods relevant to applications covered in this review. Next, we discuss specific applications of DL in oncology, including cancer origin detection, molecular subtypes identification, prognosis and survivability prediction, histological inference of genomic traits, tumour microenvironment profiling and future applications in spatial transcriptomics, metagenomics and pharmacogenomics. We conclude with an examination of current challenges and potential strategies that would enable DL to be routinely applied in clinical settings.

## Emerging deep learning methods

Covering all DL methods in detail is outside the scope of this review; rather, we provide a high-level summary of emerging DL methods in oncology. DL utilises artificial neural networks to extract non-linear, entangled and representative features from massive and high-dimensional data [1]. A deep neural network is typically constructed of millions of densely interconnected computing neurons organised into consecutive layers. Within each layer, a neuron is connected to other neurons in the layer before it, from which it receives data, and other neurons in the layer after it, to which it sends data. When presented with data, a neural network feeds each training sample, with known ground truth, to its input layer before passing the information down to all succeeding layers (usually called hidden layers). This information is then multiplied, divided, added and subtracted millions of times before it reaches the output layer, which becomes the prediction. For supervised deep learning, each pair of training sample and label is fed through a neural network while its weights and thresholds are being adjusted to get the prediction closer to the provided label. When faced with unseen (test) data, these trained weights and thresholds are frozen and used to make predictions.

### Fundamental neural network methods

There are multiple neural network-based methods, all with different advantages and applications. Multilayer perceptron (MLP), recurrent neural network (RNN) and convolutional neural network (CNN) are the most fundamental and are frequently used as building blocks for more advanced techniques. MLPs are the simplest type of neural networks, where neurons are organised in consecutive layers so that signals travel through the network in one direction (from input to output) [1]. Although MLPs can perform well for generic predictions, they are also prone to overfitting [6]. RNNs process an input sequence one element at a time, while maintaining history of all past elements in hidden ‘state vector(s)’. Output predictions are made at every element using information from the current element and also previous elements [1, 7]. RNNs are typically used for analysing sequential data such as text, speech or DNA sequences. By contrast, CNNs are designed to draw spatial relationships from image data. CNNs traverse an image and apply small feature-filter matrices, i.e. convolution filters, to extract granular features [1]. Features extracted by the last convolution layer are then used for making predictions. CNNs have also been adapted for analysis of non-image data, e.g. genomic data represented in a vector, matrix or tensor format [8]. A review by Dias and Torkamani [7] described in detail how MLPs, RNNs and CNNs operate on biomedical and genomics data. Moreover, the use of MLPs, RNNs and CNNs to assist clinicians and researchers has been proposed across multiple oncology areas, including radiotherapy [9], digital histopathology [10, 11] and clinical and genomic diagnostics [7]. While routine clinical use is still limited, some of the models have already been FDA-approved and adopted into a clinical setting, for example CNNs for the prediction of malignancy in pulmonary nodules detected by CT [12], and prostate and breast cancer diagnosis prediction using digital histopathology [13, 14].

### Advanced neural-network methods

**Fig. 2:**
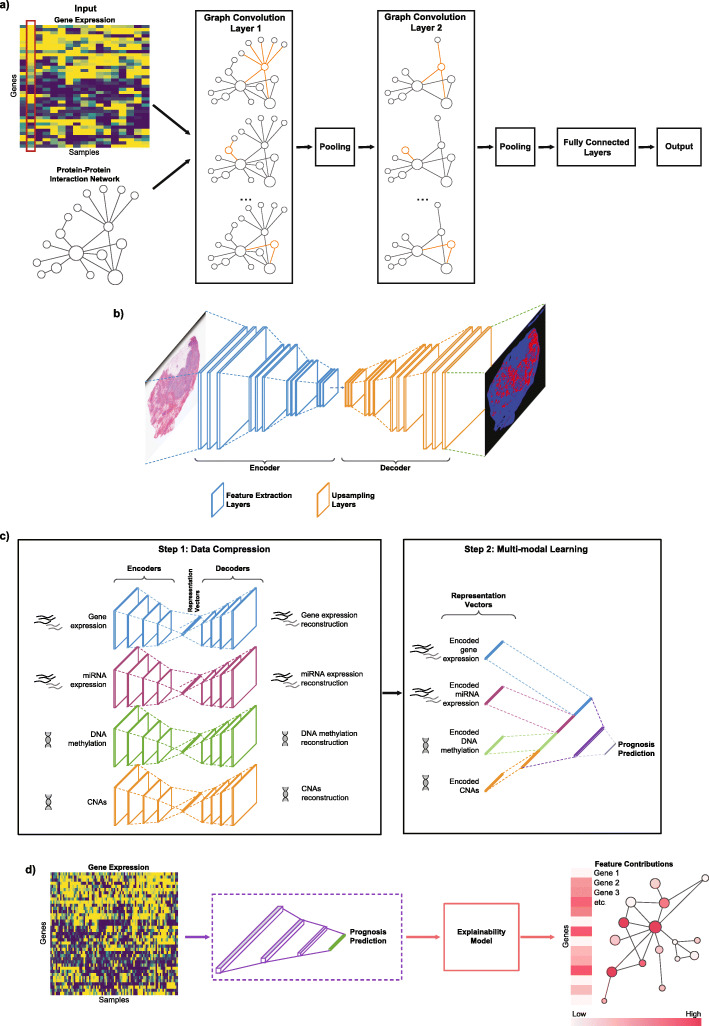
An overview of Deep Learning techniques and concepts in oncology. **a** Graph convolutional neural networks (GCNN) are designed to operate on graph-structured data. In this particular example inspired by [17–19], gene expression values (upper left panel) are represented as graph signals structured by a protein–protein interactions graph (lower left panel) that serve as inputs to GCNN. For a single sample (highlighted with red outline), each node represents one gene with its expression value assigned to the corresponding protein node, and inter-node connections represent known protein–protein interactions. GCNN methods covered in this review require a graph to be undirected. Graph convolution filters are applied on each gene to extract meaningful gene expression patterns from the gene’s neighbourhood (nodes connected by orange edges). Pooling, i.e. combining clusters of nodes, can be applied following graph convolution to obtain a coarser representation of the graph. Output of the final graph convolution/pooling layer would then be passed through fully connected layers producing GCNN’s decision. **b** Semantic segmentation is applied to image data where it assigns a class label to each pixel within an image. A semantic segmentation model usually consists of an encoder, a decoder and a softmax function. The encoder consists of feature extraction layers to ‘learn’ meaningful and granular features from the input, while the decoder learns features to generate a coloured map of major object classes in the input (through the use of the softmax function). The example shows a H&E tumour section with infiltrating lymphocyte map generated by Saltz et al. [20] DL model **c** multimodal learning allows multiple datasets representing the same underlying phenotype to be combined to increase predictive power. Multimodal learning usually starts with encoding each input modality into a representation vector of lower dimension, followed by a feature combination step to aggregate these vectors together. **d** Explainability methods take a trained neural network and mathematically quantify how each input feature influences the model’s prediction. The outputs are usually feature contribution scores, capable of explaining the most salient features that dictate the model’s predictions. In this example, each input gene is assigned a contribution score by the explainability model (colour scale indicates the influence on the model prediction). An example of gene interaction network is shown coloured by contribution scores (links between red dots represent biological connections between genes)

Graph convolutional neural networks (GCNNs) generalise CNNs beyond regular structures (Euclidean domains) to non-Euclidean domains such as graphs which have arbitrary structure. GCNNs are specifically designed to analyse graph data, e.g. using prior biological knowledge of an interconnected network of proteins with nodes representing proteins and pairwise connections representing protein–protein interactions (PPI) [15], using resources such as the STRING PPI database [16] (Fig. 2a). This enables GCNNs to incorporate known biological associations between genetic features and perceive their cooperative patterns, which have been shown to be useful in cancer diagnostics [17].

Semantic segmentation is an important CNN-based visual learning method specifically for image data (Fig. 2b). The purpose of semantic segmentation is to produce a class label for every single pixel in an image and cluster parts of an image together into each class, where the class represents an object or component of the image. Semantic segmentation models are generally supervised, i.e. they are given class labels for each pixel and are trained to detect the major ‘semantics’ for each class.

To enhance the predictive power of DL models, different data types (modalities) can be combined using multimodal learning (Fig. 2c). In clinical oncology, data modalities can include image, numeric and descriptive data. Cancer is a complex and multi-faceted disease with layers of microscopic, macroscopic and molecular features that can separately or together influence treatment responses and patient prognosis. Therefore, combining clinical data (e.g. diagnostic test results and pathology reports), medical images (e.g. histopathology and computed tomography) and different types of omics data, such as genomic, transcriptomic and proteomic profiles, may be useful. The two most important requirements for a multimodal network are the ability to create representations that contain dense meaningful features of the original input, and a mathematical method to combine representations from all modalities. There are several methods capable of performing the representative learning task, e.g. CNNs, RNNs, deep belief networks and autoencoders (AE) [21]; score-level fusion [22]; or multimodal data fusion [23]. The multimodal learning applications discussed in this review are based on AE models. In simplistic terms, AE architecture comprises of an encoder and a decoder working in tandem. The encoder is responsible for creating a representation vector of lower dimension than the input, while the decoder is responsible for reconstructing the original input using this low-dimensional vector [24]. This forces the encoder to ‘learn’ to encapsulate meaningful features from the input and has been shown to have good generalisability [24]. Moreover, it provides DL models the unique ability to readily integrate different data modalities, e.g. medical images, genomic data and clinical information, into a single ‘end-to-end optimised’ model [8].

A major challenge with implementing DL into clinical practice is the ‘black box’ nature of the models [25]. High-stake medical decisions, such as diagnosis, prognosis and treatment selection, require trustworthy and explainable decision processes. Most DL models have limited interpretability, i.e. it is very difficult to dissect a neural network and understand how millions of parameters work simultaneously. Some even argue that more interpretable models such as Decision Trees should be ultimately preferred for making medical decisions [26]. An alternative approach is explainability—mathematical quantification of how influential, or ‘salient’, the features are towards a certain prediction (Fig. 2d). This information can be used to ‘explain’ the decision-making process of a neural network model and identify features that contribute to a prediction. This knowledge can enable resolution of potential disagreements between DL models and clinicians and thus increase trust in DL systems [27]. Moreover, DL models do not always have perfect performance due to either imperfect training data (e.g. assay noise or errors in recording) or systematic errors caused by bias within DL models themselves, which can result from the training data not being representative of the population where DL is later applied [27]. In these circumstances, explainability can assist clinicians in evaluating predictions [27]. While some explainability methods were developed specifically for neural networks [28, 29], others offer a more model- and data-agnostic solution [30–33]. Excitingly, explainability methods can be used in conjunction with multi-modal learning for data integration and discovery of cross-modality insights, e.g. how cancer traits across different omics types correlate and influence each other.

Another challenge in applying DL in oncology is the requirement for large amounts of robust, well-phenotyped training data to achieve good model generalisability. Large curated ‘ground-truth’ datasets of matched genomic, histopathological and clinical outcome data are scarce beyond the publicly available datasets, such as The Cancer Genome Atlas (TCGA) [34], International Cancer Genome Consortium (ICGC) [35], Gene Expression Omnibus (GEO) [36], European Genome-Phenome Archive (EGA) [37] and Molecular Taxonomy of Breast Cancer International Consortium (METABRIC) [38]. Pre-training on abundant datasets from other domains may help overcome the challenges of limited data (a process known as transfer learning). The pre-trained neural network would then be reconfigured and trained again on data from the domain of interest. This approach usually results in a considerable reduction in computational and time resources for models training, and a significant increase in predictive performance, compared to training on small domain-specific datasets [39].

## Deep learning in oncology

A variety of DL approaches that utilise a combination of genomic, transcriptomic or histopathology data have been applied in clinical and translational oncology with the aim of enhancing patient diagnosis, prognosis and treatment selection (Fig. 1, Table 1). However, even with the emerging DL approaches, human intervention remains essential in oncology. Therefore, the goal of DL is not to outperform or replace humans, but to provide decision support tools that assist cancer researchers to study the disease and health professionals in the clinical management of people with cancer [79].

**Table 1:** Summary of deep learning methods, their relevant applications and brief technical descriptions of each DL model

Application	DL method	Reference	Description
Microscopy-based assessment of cancer	CNN	Ruy et al. [40] Nir et al. [41] Ström et al. [42] Ehteshami Bejnordi et al. [43] Vuong et al. [44] El Achi and Khoury [45]	Trained CNNs on pathology images to predict grading of prostate [40–42], breast [43], colon cancer [44] and lymphoma [45]
	CNN & explainability	Hägele et al. [46]	LRP used to assigned feature contribution for cancer grade for each pixel of WSIs
	Semantic segmentation	Poojitha and Lal Sharma [47]	A semantic segmentation technique called GAN was used to segment tissue maps for prostate cancer grade prediction
Molecular subtyping	MLP	DeepCC [48]	Gene set enrichment analysis used to transform gene expression input into functional spectra
	CNN	imCMS [49], Sirinukuwattana et al. [50], Stalhammar et al. [51], Couture et al. [52] Woerl et al. [53]	Models trained on histopathology images to classify molecular subtypes of of lung [49], colorectal [50], breast [51, 52] and bladder cancer [53]
	GCNN	Rhee et al. [18]	Utilised a hybrid GCNN model to organise input gene expression profiles into STRING PPI network [16] and predict breast cancer molecular subtypes
	Multimodal learning	Islam et al. [54]	Two CNN models used to predict breast cancer molecular subtypes from CNAs and gene expression; Outputs of the last fully connected layer of each model concatenated for a final subtype prediction
Cancer of unknown primary	MLP	Jiao et al. [55]	Model trained to predict origins of 24 cancer types using somatic mutation patterns and driver genes
	CNN	SCOPE [56], CUP-AI-Dx [57]	Both studies trained models to predict different cancer types from gene expression
	RNN & explainability	TOAD [58]	RNN-based model called Attention was trained on WSIs to predict metastasis and cancer origin; Attention algorithm reveal image regions contributing most to predictions were mostly cancer cells
Prognosis prediction	MLP	Cox-nnet [59], DeepSurv [60], RankedDeepSurv [61]	Cox regression used as the last layer of MLP models for prognosis prediction
	MLP & AEs	AECOX [62]	AE used to “compress” gene expression into low-dimensional embedding vector and used as an input for Cox-regression
	Explainability	PASNET [63], Cox-PASNET [64]	A pathway layer used between the input and the hidden layers with each node representing a known pathway; Analysis of weight differences in pathway layers reveal clinically actionable genetic traits
		MesoNet [65]	Histopathology images split into tiles and scored by survival prediction contributions; Scores used to identify top-contributing regions, reviewed by pathologists
	GCNN & explainability	Chereda et al. [19]	Combine GCNN and explainability method LRP to identify biologically and therapeutically relevant genes in predicting metastasis of breast cancer
	Explainability with multimodal learning	PAGE-Net [66]	CNN used to compress features from WSIs; Cox-PASNet used to incorporate gene pathway and provide cross-modal analysis with image features extracted by CNN
		PathME [67]	AEs used to compress features from four omics modalities, which are combined to predict survival; SHAP used to assign each omics feature survival prediction contribution score
Precision Oncology	MLP	HER2RNA [68]	Transcriptomic profiles inferred from histopathology images divided into tiles; Predictions added up for all tiles and compared with ‘ground truth’ transcriptomic profiles
	CNN	Image2TMB [69]	Ensemble of three CNNs to extract features from histopathological images at different resolutions (x5, x10 and x20); Extracted features are combined to infer TMB
		Kather et al. [70]	TCGA histopathology images used to predict mutational status of key genes, molecular subtypes and gene expression of standard biomarkers
Tumour microenvironment	MLP	Scaden [71]	Ensemble of three models with different filter sizes to predict TME composition from gene expression; Predictions from the models are averaged into a final prediction
	Explainability with MLP	MethylNet [72]	MLP and AE used to ‘compress’ CpG beta values into an embedding vector for predicting TME composition; SHAP used to assign feature contribution to each CpG site
	Semantic segmentation	Saltz et al. [20]	Semantic segmentation model used on H&E images to localise spatial heterogeneity patterns of TIL and necrosis
Spatial transcriptomics	CNN	ST-Net [73]	Images split into tiles centred on spatial transcriptomics spots; Tiles used to train a CNN to predict expression of 250 target genes
Pharmacogenomics	CNN	CDRscan [74]	Two models used to extract features from somatic mutational fingerprints and molecular profiles of drugs (cell lines); Feature vectors combined to predict efficacy of drugs based on genomic profiles
	MLP	DeepSynergy [75]	Cell line gene expression and chemical features of drugs in drug combinations used as input; Predicts ‘synergy score’ between the drug combinations and transcriptomic profiles
	GCNN	Jiang et al. [76]	Utilised graph structure to integrate protein-protein, drug-drug and drug-protein interactions to predict synergistic drug combination for specific cell lines
	Multimodal learning	DeepDR [77]	Collection of ten AEs to integrate ten drug-disease networks, which predict drug-disease associations
	CNN	DeepDTI [78]	Protein sequence and drug fingerprint as input to predict drug protein-binding sites

*AE:* autoencoder, *CNA:* copy number alterations, *CNN:* convolutional neural network, *DL:* deep learning, *GCNN:* graph convolutional neural network, *H&E:* haematoxylin and eosin, *LRP:* layer-wise relevance propagation, *MLP:* multilayer perceptron, *RNN:* recurrent neural netowrk, *SHAP:* SHapley Additive exPlanations, *TIL:* tumour-infiltrating lymphocytes, *TMB:* tumour mutational burden, *WSI:* whole slide image

### Deep learning for microscopy-based assessment of cancer

Cancers are traditionally diagnosed by histopathology or cytopathology to confirm the presence of tumour cells within a patient sample, assess markers relevant to cancer and to characterise features such as tumour type, stage and grade. This microscopy-based assessment is crucial; however, the process is relatively labour-intensive and somewhat subjective [80, 81]. A histology image viewed at high magnification (typically 20x or 40x) can reveal millions of subtle cellular features, and deep CNN models are exceptionally good at extracting features from high-resolution image data [82]. Automating cancer grading with histology-based deep CNNs has proven successful, with studies showing that performance of deep CNNs can be comparable with pathologists in grading prostate [40–42], breast [43], colon cancer [44] and lymphoma [45]. Explainability methods can enable and improve histology-based classification models by allowing pathologists to validate DL-generated predictions. For example, Hägele et al. applied the Layer-wise Relevance Propagation (LRP) [29] method on DL models classifying healthy versus cancerous tissues using whole-slide images of lung cancer [46]. The LRP algorithm assigned a relevance score for each pixel, and pixel-wise relevance scores were aggregated into cell-level scores and compared against pathologists’ annotations. These scores were then used to evaluate DL model performance and identify how multiple data biases affected the performance at cellular levels [46]. These insights allow clinician and software developers to gain insights into DL models during development and deployment phases.

In addition to classification and explainability, semantic segmentation approaches can also be applied on histopathology images to localise specific regions. One notable approach to perform semantic segmentation is to use generative adversarial networks (GANs) [47]. GAN is a versatile generative DL method comprising a pair of two neural networks: a generator and a discriminator [83]. In the context of semantic segmentation, the generator learns to label each pixel of an image to a class object (Fig. 2b), while the discriminator learns to distinguish the predicted class labels from the ground truth [84]. This ‘adversarial’ mechanism forces the generator to be as accurate as possible in localising objects so that the discriminator cannot recognise the difference between predicted and ground-truth class labels [84]. Using this approach, Poojitha and Lal Sharma trained a CNN-based generator to segment cancer tissue to ‘help’ a CNN-based classifier predict prostate cancer grading [47]. The GAN-annotated tissue maps helped the CNN classifier achieve comparable accuracy to the grading produced by anatomical pathologists, indicating DL models can detect relevant cell regions in pathology images for decision making.

### Molecular subtyping of cancers

Transcriptomic profiling can be used to assign cancers into clinically meaningful molecular subtypes that have diagnostic, prognostic or treatment selection relevance. Molecular subtypes were first described for breast cancer [85, 86], then later for other cancers including colorectal [87], ovarian cancer [88] and sarcomas [89]. Standard computational methods, such as support vector machines (SVMs) or k-nearest neighbours, used to subtype cancers can be prone to errors due to batch effects [90] and may rely only on a handful of signature genes, omitting important biological information [91–93]. Deep learning algorithms can overcome these limitations by learning patterns from the whole transcriptome. A neural network model DeepCC trained on TCGA RNA-seq colon and breast cancer data, then tested on independent gene expression microarray data showed superior accuracy, sensitivity and specificity when compared to traditional ML approaches including random forest, logistic regression, SVM and gradient boosting machine [48]. Neural networks have also been successfully applied to transcriptomic data for molecular subtyping of lung [94], gastric and ovarian cancers [95]. DL methods have the potential to be highly generalisable in profiling cancer molecular subtypes due to their ability to train on a large number of features that are generated by transcriptomic profiling. Furthermore, due to their flexibility, DL methods can incorporate prior biological knowledge to achieve improved performance. For example, Rhee et al. trained a hybrid GCNN model on expression profiles of a cancer hallmark gene set, connected in a graph using the STRING PPI network [16] to predict breast cancer molecular subtypes, PAM50 [18]. This approach outperformed other ML methods in subtype classification. Furthermore, the granular features extracted by the GCNN model naturally clustered tumours into PAM50 subtypes without relying on a classification model demonstrating that the method successfully learned the latent properties in the gene expression profiles [18].

The use of multimodal learning to integrate transcriptomic with other omics data may enable enhanced subtype predictions. A novel multimodal method using two CNN models trained separately on copy number alterations (CNAs) and gene expression before concatenating their representations for predictions was able to predict PAM50 breast cancer subtypes better than CNNs trained on individual data types [54]. As multi-omics analysis becomes increasingly popular, multimodal learning methods are expected to become more prevalent in cancer diagnostics. However, the challenges of generating multi-omic data from patient samples in the clinical setting, as opposed to samples bio-banked for research, may hinder the clinical implementation of these approaches.

Digital histopathology images are an integral part of the oncology workflow [11] and can be an alternative to transcriptomic-based methods for molecular subtyping. CNN models have been applied on haematoxylin and eosin (H&E) sections to predict molecular subtypes of lung [49], colorectal [50], breast [51, 52] and bladder cancer [53], with greater accuracy when compared to traditional ML methods.

### Diagnosing cancers of unknown primary

Determining the primary cancer site can be important during the diagnostic process, as it can be a significant indicator of how the cancer will behave clinically, and the treatment strategies are sometimes decided by the tumour origin [96, 97]. However, 3–5% of cancer cases are metastatic cancers of unknown origin, termed cancers of unknown primary (CUPs) [98, 99]. Genomic, methylation and transcriptomic profiles of metastatic tumours have unique patterns that can reveal their tissues of origin [100–102].

Traditional ML methods, such as regression and SVMs, applied to these omics data can predict tumour origin; however, they usually rely on a small subset of genes, which can be limiting in predicting a broad range of cancer types and subtypes. In contrast, DL algorithms can utilise large number of genomic and transcriptomic features. The Pan-Cancer Analysis of Whole Genomes (PCAWG) Consortium [103] used a DL model to predict the origins of 24 cancer types individually and collectively using thousands of somatic mutation features across 2 different classes (mutational distribution and driver gene and pathway features) [55]. Remarkably, the study found that driver genes and pathways are not among the most salient features, highlighting why previous efforts in panel and exome sequencing for CUP produced mixed results [104–107]. Deep learning approaches utilising transcriptome data have also shown utility in predicting tumour site of origin [56, 57]. A neural network called SCOPE, trained on whole transcriptome TCGA data, was able to predict the origins of treatment-resistant metastatic cancers, even for rare cancers such as metastatic adenoid cystic carcinoma [56]. The CUP-AI-Dx algorithm, built upon a widely used CNN model called Inception [108], achieved similar results on 32 cancer types from TCGA and ICGC [57]. As whole genome sequencing becomes increasingly available, these models show great potential for future DL methods to incorporate multiple omics features to accurately categorise tumours into clinically meaningful subtypes by their molecular features.

In addition to genomic and transcriptomic data, a new model call TOAD trained on whole slide images (WSIs) was able to simultaneously predict metastasis status and origin of 18 tumour types [58]. Moreover, the model employed an explainability method called attention [109, 110] to assign diagnostic relevance scores to image regions and revealed that regions with cancer cells contributed most to both metastasis and origin decision making [58]. These results suggested TOAD can ‘focus’ on biologically relevant image patterns and is a good candidate for clinical deployment.

### Cancer prognosis and survival

Prognosis prediction is an essential part of clinical oncology, as the expected disease path and likelihood of survival can inform treatment decisions [111]. DL applied to genomic, transcriptomic and other data types has the potential to predict prognosis and patient survival [59–62, 112]. The most common survival prediction method is the Cox proportional hazard regression model (Cox-PH) [113–115], which is a multivariate linear regression model finding correlations between survival time and predictor variables. A challenge of applying Cox-PH on genomic and transcriptomic data is its linear nature, which can potentially neglect complex and possibly nonlinear relationships between features [116]. By contrast, deep neural networks are naturally nonlinear, and in theory could excel at this task. Interestingly, many studies have incorporated Cox regression used for survival analysis into DL and trained these models on transcriptomic data for enhanced prognosis predictions [59–62, 112]. Among them, Cox-nnet was a pioneering approach that made Cox regression the output layer of neural networks, effectively using millions of deep features extracted by hidden layers as input for the Cox regression model [59]. Cox-nnet was trained on RNA-seq data from 10 TCGA cancer types and benchmarked against two variations of Cox-PH (Cox-PH and CoxBoost). Cox-nnet showed superior accuracy and was the only model able to uniquely identify important pathways including p53 signalling, endocytosis and adherens junctions [59], demonstrating that the combination of Cox-PH and neural networks has the potential to capture biological information relating to prognosis. The potential of DL was confirmed by Huang et al. [62] who found that 3 different DL versions of Cox Regression (Cox-nnet, DeepSurv [60] and AECOX [62]) outperformed Cox-PH and traditional ML models. These results suggest that DL models can provide better accuracy than traditional models in predicting prognosis by learning from complex molecular interactions using their flexible architecture.

The incorporation of biological pathways in DL has enabled the elucidation of key survival drivers among thousands of features. PASNET [63] and its Cox-regression version Cox-PASNet [64] are among the most advanced DL models in this area. Both models incorporate a pathway layer between the input and the hidden layers of the neural network, where each node of the pathway layer represents a pathway (based on pathway databases such as Reactome [117] and KEGG [118]), and the connections between the two layers represent the gene-pathway relationships. These trained pathway nodes have different weights. By analysing the weight differences across different survival groups and identifying genes connected to each node, PASNet and Cox-PASNet were able to identify clinically actionable genetic traits of glioblastoma multiforme (GBM) and ovarian cancer [63, 64]. In GBM, Cox-PASNet correctly identified PI3K cascade, a pathway highly involved in tumour proliferation, invasion and migration in GBM [119]. Cox-PASNet also correctly detected *MAPK9,* a gene strongly associated with GBM carcinogenesis and a novel potential therapeutic, as one the most influential genes [120]. The GCNN-explainability model from Chereda et al. is the latest example of incorporating molecular networks in cancer prognosis [19]. The study used gene expression profiles, structured by a PPI from Human Protein Reference Database (HPRD) [121], to predict metastasis of breast cancer samples. The explainability method, LRP [29], was then used to identify and analyse the biological relevance of the most relevant genes for predictions [19]. Pathway analysis of these genes showed that they include oncogenes, molecular-subtype-specific and therapeutically targetable genes, such as *EGFR* and *ESR1* [19].

In addition to prognosis predictions from transcriptomic data, CNN models trained on histopathology images have been used to infer survival in several cancers including brain [122], colorectal [123], renal cell [124], liver cancers [125] and mesothelioma [65]. Among them, MesoNet [65] stands out for incorporating a feature contribution explainability algorithm called CHOWDER [126] on H&E tissue sections of mesothelioma to identify that the features contributing the most to survival predictions were primarily stromal cells associated with inflammation, cellular diversity and vacuolisation [65]. The CHOWDER algorithm enabled MesoNet to utilise large H&E images as well as segment and detect important regions for survival predictions without any local annotations by pathologists [65]. These findings suggest that ‘white-box’ DL models like MesoNet could be useful companion diagnostic tools in clinical setting by assisting clinicians in identifying known and novel histological features associated with a survival outcome.

Multi-modal DL analysis integrating histopathology images and, if available, omics data has the potential to better stratify patients into prognostic groups, as well as suggest more personalised and targeted treatments. Most multi-modal prognostic studies have focussed on three aspects: individual feature extraction from a single modality, multi-modal data integration and cross-modal analysis of prognostic features. The model PAGE-Net performed these tasks by using a CNN to create representations of WSIs and Cox-PASNet [64] to extract genetic pathway information from gene expression [66]. This architecture allowed PAGE-NET to not only integrate histopathological and transcriptomic data, but also identify patterns across both modalities that cause different survival rates [66]. More interestingly, the combination of multi-modal and explainability methods is particularly promising. PathME [67] is a pioneer of this approach by bringing together representation-extraction AEs and an explainability algorithm called SHAP [31–33, 127]. The AEs captured important features from gene expression, miRNA expression, DNA methylation and CNAs for survival prediction, while SHAP scores each feature from each omic based on how relevant it is to the prediction [67]. Together, the two algorithms detected clinically relevant cross-omics features that affect survival across GBM, colorectal, breast and lung cancer [67]. The PathME methodology is cancer-agnostic, which makes it a great candidate for clinical implementations to explore actionable biomarkers in large-scale multi-omics data. Additionally, other studies [128–130] have employed Principal Component Analysis (PCA) [131] to compress gene expression, mutational signatures and methylation status into eigengene vectors [132], which were then combined with CNN-extracted histopathology features for survival predictions. While these methods could integrate histopathology data with multi-omics, they are not as explainable as PAGE-Net [66] or PathME [67] and thus less clinically suitable, as the conversion of genes into eigengenes makes exploration of cross-modality interactions challenging.

### Precision oncology

The promise of precision medicine is to use high-resolution omics data to enable optimised management and treatment of patients to improve survival. An important part of precision oncology involves understanding cancer genomics and the tumour microenvironment (TME). DL offers the potential to infer important genomic features from readily available histopathology data, as well as disentangle the complex heterogeneity of TME to enable precision oncology.

Genomic traits such as tumour mutation burden (TMB) and microsatellite instability (MSI) have been shown to be important biomarkers of immunotherapy response across cancer types [133–136]. Assessment of these traits requires sequencing (comprehensive panel, exome or whole genome), which is still expensive and is not readily available in the clinic.

Routinely used histopathological images are a potential window to genomic features and may in future prove useful for predictions of specific clinically meaningful molecular features without the need for tumour sequencing. Several CNN methods have been developed to infer TMB, MSI and other clinically relevant genomic features from H&E sections [68–70, 137]. A model called Image2TMB used ensemble learning to predict TMB in lung cancer using H&E images. Image2TMB was able to achieve the same average accuracy as large panel sequencing with significantly less variance. It also attempted to estimate TMB for each region of an image [69], which could enable studies of histological features associated with molecular heterogeneity.

Another DL model called HE2RNA used weakly supervised learning to infer gene expression from histopathology images, which were then used to infer MSI status in colorectal cancer [68]. When compared with another DL method to predict MSI directly from H&E slides [137], HE2RNA showed superior performance on both formalin-fixed paraffin-embedded (FFPE) and frozen sections, indicating a high level of robustness across tissue processing approaches.

Kather et al. [70] has also showed that CNN models trained and evaluated on TCGA H&E slides can accurately predict a range of actionable genetic alterations across multiple cancer types, including mutational status of key genes, molecular subtypes and gene expression of standard biomarkers such as hormone receptor status. While these molecular inference methods demonstrate an intriguing application of DL in histopathology, their current clinical utility is likely to be limited as features such as MSI and hormone receptor status are already part of the routine diagnostic workflows (immunohistochemistry staining for mismatch-repair proteins in colorectal and endometrial cancer or ER, PR in breast cancer). However, these studies serve as proof-of-concept, and the developed models could in future be adapted to predict clinically important molecular features that are not routinely assessed. Thus, future investigations into histopathology-based genomic inference are warranted, with the understanding that the accuracy of such DL models needs to be exceptional for them to replace current assays.

### The tumour microenvironment

The TME plays a key role in cancer progression, metastasis and response to therapy [138]. However, there remain many unknowns in the complex molecular and cellular interactions within the TME. The rise of DL in cancer research, coupled with large publicly available catalogues of genomic, transcriptomic and histopathology data, have created a strong technical framework for the use of neural networks in profiling the heterogeneity of TME.

Infiltrating immune cell populations, such as CD4+ and CD8+ T cells, are potential important biomarkers of immunotherapy response [139, 140]. Traditional ML methods can accurately estimate TME cell compositions using transcriptomic [141, 142] or methylation data [143]. However, most of these methods rely on the generation of signature Gene Expression Profiles (GEPs) or the selection of a limited number of CpG sites, biassed to previously known biomarkers. This can lead to models susceptible to noise and bias and unable to discover novel genetic biomarkers. DL methods can be trained on the whole dataset (i.e. the whole transcriptome) to identify the optimal features without relying on GEPs. Recently developed DL TME methods include Scaden [71], a transcriptomic-based neural network model, and MethylNet, a methylation-based model [72]. MethylNet also incorporated the SHAP explainability method [31–33, 127] to quantify how relevant each CpG site is for deconvolution. While these methods currently focus on showing DL models are more robust against noise, bias and batch effects compared to traditional ML models, future follow-up studies are likely to reveal additional cellular heterogeneity traits of the TME and possibly inform treatment decisions. For example, a CNN trained on H&E slides of 13 cancer types [20] showed a strong correlation between spatial tumour infiltrating lymphocytes (TIL) patterns and cellular compositions derived by CIBERSORT (a Support Vector Regression model) [141]. These models have significant clinical implications, as rapid and automated identification of the composition, amount and spatial organisation of TIL can support the clinical decision making for prognosis predictions (for example, for breast cancer) and infer treatment options, specifically immunotherapy. We expect future DL methods will further explore the integrations of histopathology and omics in profiling tumour immune landscape [144]. We also expect future DL methods to incorporate single-cell transcriptomics (scRNA-Seq) data to improve TME predictions and even infer transcriptomic profiles of individual cell types. Several DL methods have already been developed to address batch correction, normalisation, imputation, dimensionality reduction and cell annotations for scRNA-Seq cancer data [145–147]. However, these studies are still experimental and require further effort and validation to be clinically applicable [148].

### The new frontiers

An exciting new approach for studying the TME is spatial transcriptomics which allows quantification of gene expression in individual cells or regions while maintaining their positional representation, thus capturing spatial heterogeneity of gene expression at high resolution [149, 150]. Given the complexity of this data, DL approaches are well suited for its analysis and interpretation. For example, by integrating histopathology images and spatial transcriptomics, DL can predict localised gene expression from tissue slides, as demonstrated by ST-Net, a neural network capable of predicting expressions of clinically relevant genes in breast cancer using tissue spots from H&E slides [73]. As the cost of spatial transcriptomics decreases in the future, it is expected more translational applications of DL will arise, for example utilising spatial transcriptomics information for improved prognosis predictions, subtype classification and refining our understanding of tumour heterogeneity [151].

In addition, gut microbiome, i.e. metagenome, has been an emerging field and shown to play an important role in cancer treatment efficacy and outcomes [152, 153]. As more multi-omics datasets (genomics, transcriptomics, proteomics, microbiotics) are being generated, annotated and made available, we speculate that integrative analysis between these data types will help mapping omics profiles of each individual patient to the metagenome, which will unlock effective new exciting options.

Lastly, pharmacogenomics, to predict drug responses and the mechanisms of action using genomic characteristics, is an important and exciting area in precision oncology where DL methods have significant potential [154]. The increasing availability of public omics data has facilitated recent growth of DL applications in cancer pharmacogenomics [155–157]. Most common applications include therapy response and resistance (e.g. Dr.VAE [158] or CDRscan [74]), drug combination synergy (e.g. DeepSynergy [75] and Jiang et al. [76]), drug repositioning (e.g. deepDR [77]) and drug-target interactions (e.g. DeepDTI [78]). As pharmacogenomics is a highly translational field, we expect many such DL models will be applied in clinical setting in the future.

## Challenges and limitations: the road to clinical implementation

This review provides an overview of exciting potential DL applications in oncology. However, there are several challenges to the widespread implementation of DL in clinical practice. Here, we discuss challenges and limitations of DL in clinical oncology and provide our perspective for future improvements.

### Data variability

Data variability is a major challenge for applying DL to oncology. For example, in immunohistochemistry each lab may have different intensity of staining or have different qualities of staining. It is currently unclear how DL systems would deal with this inter- and intra-laboratory variability. For transcriptomic data, one of the principal difficulties is establishing the exact processing applied to generate a sequence library and processed dataset. Even properties as basic as ‘the list of human genes’ are not settled and multiple authorities publish and regularly update lists of genes, observed spliceforms, so any analysis should specify both the source and version of the gene model used. Additionally, there are a large range of data transformations (log, linear, etc.) and data normalisations (FPKM, TMM, TPM), with implementations in multiple programming languages resulting in a combinatorially large number of possible processing paths that should theoretically return the same results but without any formal process to ensure that that assumption is true.

### Paucity of public phenotypically characterised datasets

One challenge of implementing DL into clinical practice is the need for large phenotypically characterised datasets that enable development and training of DL models with good generalisation performance. High-quality cancer datasets that have undergone omics profiling are difficult to acquire in the clinical setting due to cost, sample availability and quality. In addition, clinical tumour samples can be small and are typically stored as FFPE blocks, resulting in degraded RNA and crosslinked DNA not suitable for comprehensive molecular profiling. To overcome this, explanability methods, such as SHAP, could be applied on the current DL models, that are developed in research setting, to identify the most salient features and design targeted profiling workflows suitable for clinical samples. This way, the DL models could still capture the complexity and possible non-linear gene relationships, but be retrained to make clinical predictions using only the select salient features. Multi-modal based DL models coupled with explainability could also be explored due to their potential of using features in one modality to complement missing data in another. Transfer learning can also overcome challenges of requiring large datasets by pre-training DL models from other domains. In practice, however, large data sets with thousands of samples per class are still needed for accurate predictions in the clinic, as patient outcomes are complex and there is clinical heterogeneity between patients including responses, treatment courses, comorbidities and other lifestyle factors that may impact prognosis and survival. As more data is being routinely generated and clinical information centrally collected in digital health databases, we expect to see more DL models developed for treatment response predictions as well as the general prognosis predictions. More interestingly, DL’s ability to continue learning from and become more accurate with new training samples, i.e. active learning, can significantly help pathologists reduce time spent on training histopathology data annotation. For example, a histopathology-based DL model by Saltz et al. only required pathologists to annotate a few training images at a time, and stopping the manual annotation process when the model’s performance is satisfactory [20].

Lastly, clinical data about a sample or piece of data usually do not capture all the complexities of the samples and phenotype and can be prone to incompleteness, inconsistencies and errors. A potential strategy to address this issue is to design DL models less reliant on or independent from clinical annotations, for example the MesoNet model was able to detect prognostically meaningful regions from H&E images without any pathologist-derived annotations [65].

### AI explainability and uncertainty

Finally, for DL to be implemented and accepted in the clinic, the models need to be designed to complement and enhance clinical workflows. For human experts to effectively utilise these models, they need to be not only explainable, but also capable of estimating the uncertainty in their predictions.

Over the last 5 years, research into explainable AI has accelerated. For DL to obtain regulatory approval and be used as a diagnostic tool, comprehensive studies of the biological relevance of explainability are imperative. In medical imaging, this entails validating DL-identified clinically relevant regions against pathology review, and in some cases, cross-validation with genomic features [46]. In genomics, this entails validating DL-identified relevant genetic features against those identified by conventional bioinformatics methods, for example confirming that the most discriminatory genes in predicting tissue types, as identified by SHAP, were also identified by pairwise differential expression analysis using edgeR [159] or showing that patient-specific molecular interaction networks produced in predicting metastasis status of breast cancer were not only linked to benign/malignant phenotype, but also indicative of tumour progression and therapeutic targets [19].

Furthermore, DL model’s ability to produce the ‘I don’t know’ output, when uncertain about predictions, is critical. Most DL applications covered in this review are point-estimate methods, i.e. the predictions are simply the best guess with the highest probability. In critical circumstances, overconfident predictions, e.g. predicting cancer primary site with only 40% certainty, can result in inaccurate diagnosis or cancer management decisions. Furthermore, when uncertainty estimates are too high, companion diagnostic tools should be able to abstain from making predictions and ask for medical experts’ opinion [160]. Probabilistic DL methods capable of quantifying prediction uncertainty, such as Bayesian DL [161], are great candidates to address these issues and have recently started to be applied in cancer diagnosis tasks [162–164]. We expect probabilistic models to become mainstream in oncology in the near future.

## Conclusions

In summary, DL has the potential to dramatically transformed cancer care and bring it a step closer to the promise of precision oncology. In an era where genomics is being implemented into health delivery and health data is becoming increasingly digitised, it is anticipated that artificial intelligence and DL will be used in the development, validation and implementation of decision support tools to facilitate precision oncology. In this review, we showcased a number of promising applications of DL in various areas of oncology, including digital histopathology, molecular subtyping, cancer diagnosis, prognostication, histological inference of genomic characteristics, tumour microenvironment and emerging frontiers such as spatial transcriptomics and pharmacogenomics. As the research matures, the future of applied DL in oncology will likely focus on integration of medical images and omics data using multimodal learning that can identify biologically meaningful biomarkers. Excitingly, the combination of multimodal learning and explainability can reveal novel insights. Important prerequisites of widespread adoption of DL in clinical setting are phenotypically rich data for training models and clinical validation of the biological relevance of DL-generated insights. We expect as new technologies such as single-cell sequencing, spatial transcriptomics and multiplexed imaging become more accessible, more efforts will be dedicated to improving both the quantity and quality of labelling/annotation of medical data. Finally, for DL to be accepted in routine patient care, clinical validation of explainable DL methods will play a vital role.

## Data Availability

Not applicable

## Abbreviations

AE: Autoencoder
AI: Artificial intelligence
CUP: Cancer of unknown primary
CNAs: Copy number aberrations
CNN: Convolutional neural network
Cox-PH: Cox proportional hazard regression model
DL: Deep learning
EGA: European Genome Atlas
FFPE: Formalin-fixed, paraffin-embedded
GBM: Glioblastoma multiforme
GCNN: Graph convolutional neural network
GEO: Gene Expression Omnibus
GPU: Graphical Processing Units
HPRD: Human Protein Reference Database
H&E: Haematoxylin and Eosin
ICGC: International Cancer Genome Consortium
LRP: Layer-wise Relevance Propagation
MSI: Microsatellite instability
ML: Machine learning
MLP: Multilayer perceptron
PCAWG: Pan-Cancer Analysis of Whole Genomes
PPI: Protein-protein interactions
RNA-seq: RNA sequencing
RNN: Recurrent neural network
SVM: Support vector machine
TCGA: The Cancer Genome Atlas
TIL: Tumour infiltrating lymphocytes
TME: Tumour microenvironment
TMB: Tumour mutation burden
WCNA: Weighted correlation network analysis

## References

[1] LeCun Y, Bengio Y, Hinton G (2015). Deep learning. Nature..

[2] Libbrecht MW, Noble WS (2015). Machine learning applications in genetics and genomics. Nat Rev Genet.

[3] Jones W, Alasoo K, Fishman D, Parts L (2017). Computational biology: deep learning. Skolnick J, editor. Emerg Top Life Sci.

[4] Wainberg M, Merico D, Delong A, Frey BJ (2018). Deep learning in biomedicine. Nat Biotechnol.

[5] Zou J, Huss M, Abid A, Mohammadi P, Torkamani A, Telenti A (2019). A primer on deep learning in genomics. Nat Genet.

[6] Montesinos-López OA, Montesinos-López A, Pérez-Rodríguez P, Barrón-López JA, Martini JWR, Fajardo-Flores SB (2021). A review of deep learning applications for genomic selection. BMC Genomics.

[7] Dias R, Torkamani A (2019). Artificial intelligence in clinical and genomic diagnostics. Genome Med.

[8] Eraslan G, Avsec Ž, Gagneur J, Theis FJ (2019). Deep learning: new computational modelling techniques for genomics. Nat Rev Genet.

[9] Huynh E, Hosny A, Guthier C, Bitterman DS, Petit SF, Haas-Kogan DA (2020). Artificial intelligence in radiation oncology. Nat Rev Clin Oncol.

[10] Bera K, Schalper KA, Rimm DL, Velcheti V, Madabhushi A (2019). Artificial intelligence in digital pathology—new tools for diagnosis and precision oncology. Nat Rev Clin Oncol.

[11] Huss R, Coupland SE (2020). Software-assisted decision support in digital histopathology. J Pathol.

[12] Massion PP, Antic S, Ather S, Arteta C, Brabec J, Chen H (2020). Assessing the accuracy of a deep learning method to risk stratify indeterminate pulmonary nodules. Am J Respir Crit Care Med.

[13] Kanan C, Sue J, Grady L, Fuchs TJ, Chandarlapaty S, Reis-Filho JS, Salles PGO, da Silva LM, Ferreira CG, Pereira EM (2020). Independent validation of paige prostate: assessing clinical benefit of an artificial intelligence tool within a digital diagnostic pathology laboratory workflow. J Clin Oncol.

[14] Silva LM, Pereira EM, Salles PG, Godrich R, Ceballos R, Kunz JD (2021). Independent real-world application of a clinical-grade automated prostate cancer detection system. J Pathol.

[15] Schulte-Sasse R, Budach S, Hnisz D, Marsico A (2019). Graph convolutional networks improve the prediction of cancer driver genes. Artif Neural Netw Mach Learn – ICANN 2019 [Internet].

[16] Szklarczyk D, Franceschini A, Wyder S, Forslund K, Heller D, Huerta-Cepas J (2015). STRING v10: protein–protein interaction networks, integrated over the tree of life. Nucleic Acids Res.

[17] Ramirez R, Chiu Y-C, Hererra A, Mostavi M, Ramirez J, Chen Y, Huang Y, Jin YF (2020). Classification of cancer types using graph convolutional neural networks. Front Phys.

[18] Rhee S, Seo S, Kim S (2018). Hybrid approach of relation network and localized graph convolutional filtering for breast cancer subtype classification. Proc Twenty-Seventh Int Jt Conf Artif Intell [Internet].

[19] Chereda H, Bleckmann A, Menck K, Perera-Bel J, Stegmaier P, Auer F (2021). Explaining decisions of graph convolutional neural networks: patient-specific molecular subnetworks responsible for metastasis prediction in breast cancer. Genome Med.

[20] Saltz J, Gupta R, Hou L, Kurc T, Singh P, Nguyen V (2018). Spatial organization and molecular correlation of tumor-infiltrating lymphocytes using deep learning on pathology images. Cell Rep.

[21] Gao J, Li P, Chen Z, Zhang J (2020). A survey on deep learning for multimodal data fusion. Neural Comput.

[22] Sun D, Wang M, Li A (2019). A multimodal deep neural network for human breast cancer prognosis prediction by integrating multi-dimensional data. IEEE/ACM Trans Comput Biol Bioinform.

[23] Cheerla A, Gevaert O (2019). Deep learning with multimodal representation for pancancer prognosis prediction. Bioinformatics..

[24] Tschannen M, Bachem O, Lucic M. Recent advances in autoencoder-based representation learning. ArXiv181205069 Cs Stat [Internet]. 2018; [cited 2020 Apr 21]; Available from: http://arxiv.org/abs/1812.05069.

[25] Kelly CJ, Karthikesalingam A, Suleyman M, Corrado G, King D (2019). Key challenges for delivering clinical impact with artificial intelligence. BMC Med.

[26] Rudin C (2019). Stop explaining black box machine learning models for high stakes decisions and use interpretable models instead. Nat Mach Intell.

[27] Amann J, Blasimme A, Vayena E, Frey D, Madai VI, The Precise4Q consortium (2020). Explainability for artificial intelligence in healthcare: a multidisciplinary perspective. BMC Med Inform Decis Mak.

[28] Shrikumar A, Greenside P, Kundaje A. Learning important features through propagating activation differences. ArXiv170402685 Cs [Internet]. 2019; [cited 2020 Apr 20]; Available from: http://arxiv.org/abs/1704.02685.

[29] Bach S, Binder A, Montavon G, Klauschen F, Müller K-R, Samek W. On pixel-wise explanations for non-linear classifier decisions by layer-wise relevance propagation. Suarez OD, editor. PLoS One. 2015;10:e0130140. 10.1371/journal.pone.0130140 PMC4498753 26161953

[30] Ribeiro MT, Singh S, Guestrin C (2016). “Why Should I Trust You?”: explaining the predictions of any classifier. Proc 22nd ACM SIGKDD Int Conf Knowl Discov Data Min [Internet].

[31] Lundberg SM, Lee S-I. A unified approach to interpreting model predictions. NIPS17 Proc 31st. Int Conf Neural Inf Process Syst Curran Associates Inc. 2017;30:4768–77.

[32] Erion G, Janizek JD, Sturmfels P, Lundberg S, Lee S-I. Learning explainable models using attribution priors. ArXiv190610670 Cs Stat [Internet]. 2019; [cited 2020 Jun 22]; Available from: http://arxiv.org/abs/1906.10670.

[33] Lundberg SM, Erion G, Chen H, DeGrave A, Prutkin JM, Nair B, Katz R, Himmelfarb J, Bansal N, Lee SI (2020). From local explanations to global understanding with explainable AI for trees. Nat Mach Intell.

[34] Weinstein JN, Collisson EA, Mills GB, Shaw KRM, Ozenberger BA, The Cancer Genome Atlas Research Network (2013). The Cancer Genome Atlas Pan-Cancer analysis project. Nat Genet.

[35] The International Cancer Genome Consortium (2010). International network of cancer genome projects. Nature..

[36] Edgar R (2002). Gene Expression Omnibus: NCBI gene expression and hybridization array data repository. Nucleic Acids Res.

[37] Lappalainen I, Almeida-King J, Kumanduri V, Senf A, Spalding JD, ur-Rehman S (2015). The European Genome-phenome Archive of human data consented for biomedical research. Nat Genet.

[38] Curtis C, Shah SP, Chin S-F, Turashvili G, Rueda OM, METABRIC Group (2012). The genomic and transcriptomic architecture of 2,000 breast tumours reveals novel subgroups. Nature..

[39] Zhuang F, Qi Z, Duan K, Xi D, Zhu Y, Zhu H, et al. A comprehensive survey on transfer learning. ArXiv191102685 Cs Stat [Internet]. 2020; [cited 2020 Dec 6]; Available from: http://arxiv.org/abs/1911.02685.

[40] Ryu HS, Jin M-S, Park JH, Lee S, Cho J, Oh S (2019). Automated gleason scoring and tumor quantification in prostate core needle biopsy images using deep neural networks and its comparison with pathologist-based assessment. Cancers..

[41] Nir G, Karimi D, Goldenberg SL, Fazli L, Skinnider BF, Tavassoli P (2019). Comparison of artificial intelligence techniques to evaluate performance of a classifier for automatic grading of prostate cancer from digitized histopathologic images. JAMA Netw Open.

[42] Ström P, Kartasalo K, Olsson H, Solorzano L, Delahunt B, Berney DM (2020). Artificial intelligence for diagnosis and grading of prostate cancer in biopsies: a population-based, diagnostic study. Lancet Oncol.

[43] Ehteshami Bejnordi B, Mullooly M, Pfeiffer RM, Fan S, Vacek PM, Weaver DL (2018). Using deep convolutional neural networks to identify and classify tumor-associated stroma in diagnostic breast biopsies. Mod Pathol.

[44] Vuong TLT, Lee D, Kwak JT, Kim K (2020). Multi-task deep learning for colon cancer grading. 2020 Int Conf Electron Inf Commun ICEIC [Internet].

[45] El Achi HE, Khoury JD (2020). Artificial intelligence and digital microscopy applications in diagnostic hematopathology. Cancers..

[46] Hägele M, Seegerer P, Lapuschkin S, Bockmayr M, Samek W, Klauschen F (2020). Resolving challenges in deep learning-based analyses of histopathological images using explanation methods. Sci Rep.

[47] Poojitha UP, Lal SS (2019). Hybrid unified deep learning network for highly precise gleason grading of prostate cancer. 2019 41st Annu Int Conf IEEE Eng Med Biol Soc EMBC [Internet].

[48] Gao F, Wang W, Tan M, Zhu L, Zhang Y, Fessler E (2019). DeepCC: a novel deep learning-based framework for cancer molecular subtype classification. Oncogenesis..

[49] Yu K-H, Wang F, Berry GJ, Ré C, Altman RB, Snyder M (2020). Classifying non-small cell lung cancer types and transcriptomic subtypes using convolutional neural networks. J Am Med Inform Assoc.

[50] Sirinukunwattana K, Domingo E, Richman SD, Redmond KL, Blake A, Verrill C (2020). Image-based consensus molecular subtype (imCMS) classification of colorectal cancer using deep learning. Gut.

[51] Stålhammar G, Fuentes Martinez N, Lippert M, Tobin NP, Mølholm I, Kis L, Rosin G, Rantalainen M, Pedersen L, Bergh J, Grunkin M, Hartman J (2016). Digital image analysis outperforms manual biomarker assessment in breast cancer. Mod Pathol.

[52] Couture HD, Williams LA, Geradts J, Nyante SJ, Butler EN, Marron JS (2018). Image analysis with deep learning to predict breast cancer grade, ER status, histologic subtype, and intrinsic subtype. NPJ Breast Cancer.

[53] Woerl A-C, Eckstein M, Geiger J, Wagner DC, Daher T, Stenzel P (2020). Deep Learning Predicts Molecular Subtype of Muscle-invasive bladder cancer from conventional histopathological slides. Eur Urol.

[54] Md MI, Huang S, Ajwad R, Chi C, Wang Y, Hu P (2020). An integrative deep learning framework for classifying molecular subtypes of breast cancer. Comput Struct Biotechnol J.

[55] Jiao W, Atwal G, Polak P, Karlic R, PCAWG Tumor Subtypes and Clinical Translation Working Group, PCAWG Consortium (2020). A deep learning system accurately classifies primary and metastatic cancers using passenger mutation patterns. Nat Commun.

[56] Grewal JK, Tessier-Cloutier B, Jones M, Gakkhar S, Ma Y, Moore R, Mungall AJ, Zhao Y, Taylor MD, Gelmon K, Lim H, Renouf D, Laskin J, Marra M, Yip S, Jones SJM (2019). Application of a neural network whole transcriptome–based pan-cancer method for diagnosis of primary and metastatic cancers. JAMA Netw Open.

[57] Zhao Y, Pan Z, Namburi S, Pattison A, Posner A, Balachander S (2020). CUP-AI-Dx: A tool for inferring cancer tissue of origin and molecular subtype using RNA gene-expression data and artificial intelligence. EBioMedicine..

[58] Lu MY, Chen TY, Williamson DFK, Zhao M, Shady M, Lipkova J (2021). AI-based pathology predicts origins for cancers of unknown primary. Nature..

[59] Ching T, Zhu X, Garmire LX. Cox-nnet: an artificial neural network method for prognosis prediction of high-throughput omics data. Markowetz F, editor. PLoS Comput Biol. 2018;14:e1006076. 10.1371/journal.pcbi.1006076 PMC5909924 29634719

[60] Katzman JL, Shaham U, Cloninger A, Bates J, Jiang T, Kluger Y (2018). DeepSurv: personalized treatment recommender system using a Cox proportional hazards deep neural network. BMC Med Res Methodol.

[61] Jing B, Zhang T, Wang Z, Jin Y, Liu K, Qiu W (2019). A deep survival analysis method based on ranking. Artif Intell Med.

[62] Huang Z, Johnson TS, Han Z, Helm B, Cao S, Zhang C (2020). Deep learning-based cancer survival prognosis from RNA-seq data: approaches and evaluations. BMC Med Genet.

[63] Hao J, Kim Y, Kim T-K, Kang M (2018). PASNet: pathway-associated sparse deep neural network for prognosis prediction from high-throughput data. BMC Bioinformatics.

[64] Hao J, Kim Y, Mallavarapu T, Oh JH, Kang M (2019). Interpretable deep neural network for cancer survival analysis by integrating genomic and clinical data. BMC Med Genet.

[65] Courtiol P, Maussion C, Moarii M, Pronier E, Pilcer S, Sefta M (2019). Deep learning-based classification of mesothelioma improves prediction of patient outcome. Nat Med.

[66] Hao J, Kosaraju SC, Tsaku NZ, Song DH, Kang M (2019). PAGE-Net: interpretable and integrative deep learning for survival analysis using histopathological images and genomic data. Biocomput 2020 [Internet].

[67] Lemsara A, Ouadfel S, Fröhlich H (2020). PathME: pathway based multi-modal sparse autoencoders for clustering of patient-level multi-omics data. BMC Bioinformatics.

[68] Schmauch B, Romagnoni A, Pronier E, Saillard C, Maillé P, Calderaro J (2020). A deep learning model to predict RNA-Seq expression of tumours from whole slide images. Nat Commun.

[69] Jain MS, Massoud TF (2020). Predicting tumour mutational burden from histopathological images using multiscale deep learning. Nat Mach Intell.

[70] Kather JN, Heij LR, Grabsch HI, Loeffler C, Echle A, Muti HS, Krause J, Niehues JM, Sommer KAJ, Bankhead P, Kooreman LFS, Schulte JJ, Cipriani NA, Buelow RD, Boor P, Ortiz-Brüchle N, Hanby AM, Speirs V, Kochanny S, Patnaik A, Srisuwananukorn A, Brenner H, Hoffmeister M, van den Brandt PA, Jäger D, Trautwein C, Pearson AT, Luedde T (2020). Pan-cancer image-based detection of clinically actionable genetic alterations. Nat Can.

[71] Menden K, Marouf M, Oller S, Dalmia A, Magruder DS, Kloiber K, et al. Deep learning–based cell composition analysis from tissue expression profiles. Sci Adv [Internet]. 2020;6 Available from: https://advances.sciencemag.org/content/6/30/eaba2619. 10.1126/sciadv.aba2619 PMC7439569 32832661

[72] Levy JJ, Titus AJ, Petersen CL, Chen Y, Salas LA, Christensen BC (2020). MethylNet: an automated and modular deep learning approach for DNA methylation analysis. BMC Bioinformatics.

[73] He B, Bergenstråhle L, Stenbeck L, Abid A, Andersson A, Borg Å (2020). Integrating spatial gene expression and breast tumour morphology via deep learning. Nat Biomed Eng.

[74] Chang Y, Park H, Yang H-J, Lee S, Lee K-Y, Kim TS (2018). Cancer Drug Response Profile scan (CDRscan): a deep learning model that predicts drug effectiveness from cancer genomic signature. Sci Rep.

[75] Preuer K, Lewis RPI, Hochreiter S, Bender A, Bulusu KC, Klambauer G (2018). DeepSynergy: predicting anti-cancer drug synergy with Deep Learning. Wren J, editor. Bioinformatics..

[76] Jiang P, Huang S, Fu Z, Sun Z, Lakowski TM, Hu P (2020). Deep graph embedding for prioritizing synergistic anticancer drug combinations. Comput Struct Biotechnol J.

[77] Zeng X, Zhu S, Liu X, Zhou Y, Nussinov R, Cheng F. deepDR: a network-based deep learning approach to in silico drug repositioning. Cowen L, editor. Bioinformatics. 2019;35:5191–5198. 10.1093/bioinformatics/btz418 PMC6954645 31116390

[78] Wen M, Zhang Z, Niu S, Sha H, Yang R, Yun Y, et al. Deep-learning-based drug−target interaction prediction. J Proteome Res. 2017;16(4):1401–9. 10.1021/acs.jproteome.6b00618 28264154

[79] Walsh S, de Jong EEC, van Timmeren JE, Ibrahim A, Compter I, Peerlings J, Sanduleanu S, Refaee T, Keek S, Larue RTHM, van Wijk Y, Even AJG, Jochems A, Barakat MS, Leijenaar RTH, Lambin P (2019). Decision support systems in oncology. JCO Clin Cancer Inform.

[80] Gurcan M, Lozanski G, Pennell M, Shana′Ah A, Zhao W, Gewirtz A (2013). Inter-reader variability in follicular lymphoma grading: conventional and digital reading. J Pathol Inform.

[81] Rabe K, Snir OL, Bossuyt V, Harigopal M, Celli R, Reisenbichler ES (2019). Interobserver variability in breast carcinoma grading results in prognostic stage differences. Hum Pathol.

[82] Maggiori E, Tarabalka Y, Charpiat G, Alliez P (2017). High-resolution image classification with convolutional networks. 2017 IEEE Int Geosci Remote Sens Symp IGARSS [Internet]..

[83] Goodfellow IJ, Pouget-Abadie J, Mirza M, Xu B, Warde-Farley D, Ozair S, et al. Generative adversarial networks. ArXiv14062661 Cs Stat [Internet]. 2014; [cited 2021 Apr 27]; Available from: http://arxiv.org/abs/1406.2661.

[84] Luc P, Couprie C, Chintala S, Verbeek J. Semantic segmentation using adversarial networks. ArXiv161108408 Cs [Internet]. 2016; [cited 2021 Aug 12]; Available from: http://arxiv.org/abs/1611.08408.

[85] Sorlie T, Perou CM, Tibshirani R, Aas T, Geisler S, Johnsen H (2001). Gene expression patterns of breast carcinomas distinguish tumor subclasses with clinical implications. Proc Natl Acad Sci.

[86] Yersal O (2014). Biological subtypes of breast cancer: prognostic and therapeutic implications. World J Clin Oncol.

[87] Komor MA, Bosch LJ, Bounova G, Bolijn AS, Delis-van Diemen PM, Rausch C (2018). Consensus molecular subtype classification of colorectal adenomas: CMS classification of colorectal adenomas. J Pathol.

[88] Tothill RW, Tinker AV, George J, Brown R, Fox SB, Lade S, Johnson DS, Trivett MK, Etemadmoghadam D, Locandro B, Traficante N, Fereday S, Hung JA, Chiew YE, Haviv I, Gertig D, deFazio A, Bowtell DDL, Australian Ovarian Cancer Study Group (2008). Novel molecular subtypes of serous and endometrioid ovarian cancer linked to clinical outcome. Clin Cancer Res.

[89] Jain S, Xu R, Prieto VG, Lee P (2010). Molecular classification of soft tissue sarcomas and its clinical applications. Int J Clin Exp.

[90] Leek JT, Scharpf RB, Bravo HC, Simcha D, Langmead B, Johnson WE (2010). Tackling the widespread and critical impact of batch effects in high-throughput data. Nat Rev Genet.

[91] Haury A-C, Gestraud P, Vert J-P. The influence of feature selection methods on accuracy, stability and interpretability of molecular signatures. Teh M-T, editor. PLoS One. 2011;6:e28210. 10.1371/journal.pone.0028210 PMC3244389 22205940

[92] Kela I, Ein-Dor L, Getz G, Givol D, Domany E (2005). Outcome signature genes in breast cancer: is there a unique set?. Breast Cancer Res.

[93] Drier Y, Domany E (2011). Do two machine-learning based prognostic signatures for breast cancer capture the same biological processes? El-Rifai W, editor. PLoS One.

[94] Hu F, Zhou Y, Wang Q, Yang Z, Shi Y, Chi Q. Gene expression classification of lung adenocarcinoma into molecular subtypes. IEEE/ACM Trans Comput Biol Bioinform. 2020;17:1187–97. 10.1109/TCBB.2019.2905553 30892233

[95] Wang K, Duan X, Gao F, Wang W, Liu L, Wang X. Dissecting cancer heterogeneity based on dimension reduction of transcriptomic profiles using extreme learning machines. Wong K-K, editor. PLoS One. 2018;13:e0203824. 10.1371/journal.pone.0203824 PMC6138406 30216380

[96] Varadhachary GR, Abbruzzese JL, Lenzi R (2004). Diagnostic strategies for unknown primary cancer. Cancer..

[97] Greco FA (2013). Molecular diagnosis of the tissue of origin in cancer of unknown primary site: useful in patient management. Curr Treat Options in Oncol.

[98] Pavlidis N, Pentheroudakis G (2012). Cancer of unknown primary site. Lancet.

[99] Varadhachary GR, Raber MN (2014). Cancer of unknown primary site. N Engl J Med.

[100] Kandoth C, McLellan MD, Vandin F, Ye K, Niu B, Lu C (2013). Mutational landscape and significance across 12 major cancer types. Nature..

[101] Lawrence MS, Stojanov P, Polak P, Kryukov GV, Cibulskis K, Sivachenko A (2013). Mutational heterogeneity in cancer and the search for new cancer-associated genes. Nature..

[102] Ciriello G, Miller ML, Aksoy BA, Senbabaoglu Y, Schultz N, Sander C (2013). Emerging landscape of oncogenic signatures across human cancers. Nat Genet.

[103] The ICGC/TCGA Pan-Cancer Analysis of Whole Genomes Consortium (2020). Pan-cancer analysis of whole genomes. Nature..

[104] Chen Y, Sun J, Huang L-C, Xu H, Zhao Z (2015). Classification of cancer primary sites using machine learning and somatic mutations. Biomed Res Int.

[105] Tothill RW, Li J, Mileshkin L, Doig K, Siganakis T, Cowin P (2013). Massively-parallel sequencing assists the diagnosis and guided treatment of cancers of unknown primary: NGS in cancers of unknown primary. J Pathol.

[106] Soh KP, Szczurek E, Sakoparnig T, Beerenwinkel N (2017). Predicting cancer type from tumour DNA signatures. Genome Med.

[107] Marquard AM, Birkbak NJ, Thomas CE, Favero F, Krzystanek M, Lefebvre C (2015). TumorTracer: a method to identify the tissue of origin from the somatic mutations of a tumor specimen. BMC Med Genet.

[108] Szegedy C, Liu W, Jia Y, Sermanet P, Reed S, Anguelov D (2015). Going deeper with convolutions. 2015 IEEE Conf Comput Vis Pattern Recognit CVPR [Internet].

[109] Ilse M, Tomczak JM, Welling M. Attention-based deep multiple instance learning. arXiv:1802.04712 Cs [Internet]. 2018. [cited 2021 Sep 17]. Available from https://arxiv.org/abs/1802.04712.

[110] Lu MY, Williamson DFK, Chen TY, Chen RJ, Barbieri M, Mahmood F. Data-efficient and weakly supervised computational pathology on whole-slide images. Nat Biomed Eng [Internet]. 2021; [cited 2021 May 10]; Available from: http://www.nature.com/articles/s41551-020-00682-w. 10.1038/s41551-020-00682-w PMC8711640 33649564

[111] Nair M, Sandhu S, Sharma A (2014). Prognostic and predictive biomarkers in cancer. Curr Cancer Drug Targets.

[112] Lai Y-H, Chen W-N, Hsu T-C, Lin C, Tsao Y, Wu S (2020). Overall survival prediction of non-small cell lung cancer by integrating microarray and clinical data with deep learning. Sci Rep.

[113] Cox DR (2020). Regression Models and Life-Tables.

[114] Ahmed FE, Vos PW, Holbert D (2007). Modeling survival in colon cancer: a methodological review. Mol Cancer.

[115] de O Ferraz R, Moreira-Filho D de C (2017). Survival analysis of women with breast cancer: competing risk models. Ciênc Saúde Coletiva.

[116] Solvang HK, Lingjærde OC, Frigessi A, Børresen-Dale A-L, Kristensen VN (2011). Linear and non-linear dependencies between copy number aberrations and mRNA expression reveal distinct molecular pathways in breast cancer. BMC Bioinformatics.

[117] Fabregat A, Jupe S, Matthews L, Sidiropoulos K, Gillespie M, Garapati P, Haw R, Jassal B, Korninger F, May B, Milacic M, Roca CD, Rothfels K, Sevilla C, Shamovsky V, Shorser S, Varusai T, Viteri G, Weiser J, Wu G, Stein L, Hermjakob H, D’Eustachio P (2018). The Reactome Pathway Knowledgebase. Nucleic Acids Res.

[118] Kanehisa M, Furumichi M, Tanabe M, Sato Y, Morishima K (2017). KEGG: new perspectives on genomes, pathways, diseases and drugs. Nucleic Acids Res.

[119] Weber GL, Parat M-O, Binder ZA, Gallia GL, Riggins GJ (2011). Abrogation of PIK3CA or PIK3R1 reduces proliferation, migration, and invasion in glioblastoma multiforme cells. Oncotarget..

[120] Brahm CG, Walenkamp AME, Linde MEV, Verheul HMW, Stephan R, Fehrmann N. Identification of novel therapeutic targets in glioblastoma with functional genomic mRNA profiling. J Clin Oncol [Internet]. 2017;35 Available from: https://ascopubs.org/doi/10.1200/JCO.2017.35.15_suppl.2018.

[121] Keshava Prasad TS, Goel R, Kandasamy K, Keerthikumar S, Kumar S, Mathivanan S (2009). Human protein reference database--2009 update. Nucleic Acids Res.

[122] Zadeh Shirazi A, Fornaciari E, Bagherian NS, Ebert LM, Koszyca B, Gomez GA. DeepSurvNet: deep survival convolutional network for brain cancer survival rate classification based on histopathological images. Med Biol Eng Comput [Internet]. 2020; [cited 2020 Apr 6]; Available from: http://link.springer.com/10.1007/s11517-020-02147-3. 10.1007/s11517-020-02147-3 PMC7188709 32124225

[123] Bychkov D, Linder N, Turkki R, Nordling S, Kovanen PE, Verrill C (2018). Deep learning based tissue analysis predicts outcome in colorectal cancer. Sci Rep.

[124] Tabibu S, Vinod PK, Jawahar CV (2019). Pan-renal cell carcinoma classification and survival prediction from histopathology images using deep learning. Sci Rep.

[125] Saillard C, Schmauch B, Laifa O, Moarii M, Toldo S, Zaslavskiy M, et al. Predicting survival after hepatocellular carcinoma resection using deep-learning on histological slides. Hepatology. 2020;72(6):2000–13. 10.1002/hep.31207 32108950

[126] Courtiol P, Tramel EW, Sanselme M, Wainrib G. Classification and disease localization in histopathology using only global labels: a weakly-supervised approach. ArXiv180202212 Cs Stat [Internet]. 2020; [cited 2020 Apr 9]; Available from: http://arxiv.org/abs/1802.02212.

[127] Lundberg SM, Nair B, Vavilala MS, Horibe M, Eisses MJ, Adams T (2018). Explainable machine-learning predictions for the prevention of hypoxaemia during surgery. Nat Biomed Eng.

[128] Shao W, Cheng J, Sun L, Han Z, Feng Q, Zhang D, Frangi AF, Schnabel JA, Davatzikos C, Alberola-López C, Fichtinger G (2018). Ordinal multi-modal feature selection for survival analysis of early-stage renal cancer. Med Image Comput Comput Assist Interv – MICCAI 2018 [Internet].

[129] Ning Z, Pan W, Chen Y, Xiao Q, Zhang X, Luo J, et al. Integrative analysis of cross-modal features for the prognosis prediction of clear cell renal cell carcinoma. Schwartz R, editor. Bioinformatics. 2020;36(9):2888–95. 10.1093/bioinformatics/btaa056 31985775

[130] Shao W, Huang K, Han Z, Cheng J, Cheng L, Wang T, Sun L, Lu Z, Zhang J, Zhang D (2020). Integrative analysis of pathological images and multi-dimensional genomic data for early-stage cancer prognosis. IEEE Trans Med Imaging.

[131] Makiewicz A, Ratajczak W. Principal Components Analysis (PCA). Computers & Geosciences. 1993;19:303–42.

[132] Langfelder P, Horvath S (2008). WGCNA: an R package for weighted correlation network analysis. BMC Bioinformatics.

[133] Samstein RM, Lee C-H, Shoushtari AN, Hellmann MD, Shen R, Janjigian YY (2019). Tumor mutational load predicts survival after immunotherapy across multiple cancer types. Nat Genet.

[134] Riviere P, Goodman AM, Okamura R, Barkauskas DA, Whitchurch TJ, Lee S, Khalid N, Collier R, Mareboina M, Frampton GM, Fabrizio D, Sharabi AB, Kato S, Kurzrock R (2020). High tumor mutational burden correlates with longer survival in immunotherapy-naïve patients with diverse cancers. Mol Cancer Ther.

[135] Bao X, Zhang H, Wu W, Cheng S, Dai X, Zhu X (2020). Analysis of the molecular nature associated with microsatellite status in colon cancer identifies clinical implications for immunotherapy. J Immunother Cancer.

[136] Cortes-Ciriano I, Lee S, Park W-Y, Kim T-M, Park PJ (2017). A molecular portrait of microsatellite instability across multiple cancers. Nat Commun.

[137] Kather JN, Pearson AT, Halama N, Jäger D, Krause J, Loosen SH (2019). Deep learning can predict microsatellite instability directly from histology in gastrointestinal cancer. Nat Med.

[138] Runa F, Hamalian S, Meade K, Shisgal P, Gray PC, Kelber JA (2017). Tumor microenvironment heterogeneity: challenges and opportunities. Curr Mol Biol Rep.

[139] Borst J, Ahrends T, Bąbała N, Melief CJM, Kastenmüller W (2018). CD4+ T cell help in cancer immunology and immunotherapy. Nat Rev Immunol.

[140] Tumeh PC, Harview CL, Yearley JH, Shintaku IP, Taylor EJM, Robert L (2014). PD-1 blockade induces responses by inhibiting adaptive immune resistance. Nature..

[141] Newman AM, Liu CL, Green MR, Gentles AJ, Feng W, Xu Y (2015). Robust enumeration of cell subsets from tissue expression profiles. Nat Methods.

[142] Newman AM, Steen CB, Liu CL, Gentles AJ, Chaudhuri AA, Scherer F (2019). Determining cell type abundance and expression from bulk tissues with digital cytometry. Nat Biotechnol.

[143] Chakravarthy A, Furness A, Joshi K, Ghorani E, Ford K, Ward MJ (2018). Pan-cancer deconvolution of tumour composition using DNA methylation. Nat Commun.

[144] Klauschen F, Müller K-R, Binder A, Bockmayr M, Hägele M, Seegerer P, Wienert S, Pruneri G, de Maria S, Badve S, Michiels S, Nielsen TO, Adams S, Savas P, Symmans F, Willis S, Gruosso T, Park M, Haibe-Kains B, Gallas B, Thompson AM, Cree I, Sotiriou C, Solinas C, Preusser M, Hewitt SM, Rimm D, Viale G, Loi S, Loibl S, Salgado R, Denkert C, International Immuno-Oncology Biomarker Working Group (2018). Scoring of tumor-infiltrating lymphocytes: from visual estimation to machine learning. Semin Cancer Biol.

[145] Lopez R, Regier J, Cole MB, Jordan MI, Yosef N (2018). Deep generative modeling for single-cell transcriptomics. Nat Methods.

[146] Amodio M, van Dijk D, Srinivasan K, Chen WS, Mohsen H, Moon KR (2019). Exploring single-cell data with deep multitasking neural networks. Nat Methods.

[147] Deng Y, Bao F, Dai Q, Wu LF, Altschuler SJ (2019). Scalable analysis of cell-type composition from single-cell transcriptomics using deep recurrent learning. Nat Methods.

[148] Fan J, Slowikowski K, Zhang F (2020). Single-cell transcriptomics in cancer: computational challenges and opportunities. Exp Mol Med.

[149] Ståhl PL, Salmén F, Vickovic S, Lundmark A, Navarro JF, Magnusson J (2016). Visualization and analysis of gene expression in tissue sections by spatial transcriptomics. Science..

[150] Gerlinger M, Rowan AJ, Horswell S, Larkin J, Endesfelder D, Gronroos E (2012). Intratumor heterogeneity and branched evolution revealed by multiregion sequencing. N Engl J Med.

[151] Yoosuf N, Navarro JF, Salmén F, Ståhl PL, Daub CO (2020). Identification and transfer of spatial transcriptomics signatures for cancer diagnosis. Breast Cancer Res.

[152] Vivarelli S, Salemi R, Candido S, Falzone L, Santagati M, Stefani S (2019). Gut microbiota and cancer: from pathogenesis to therapy. Cancers..

[153] Cammarota G, Ianiro G, Ahern A, Carbone C, Temko A, Claesson MJ (2020). Gut microbiome, big data and machine learning to promote precision medicine for cancer. Nat Rev Gastroenterol Hepatol.

[154] Relling MV, Evans WE (2015). Pharmacogenomics in the clinic. Nature..

[155] Adam G, Rampášek L, Safikhani Z, Smirnov P, Haibe-Kains B, Goldenberg A (2020). Machine learning approaches to drug response prediction: challenges and recent progress. NPJ Precis Oncol.

[156] Kalinin AA, Higgins GA, Reamaroon N, Soroushmehr S, Allyn-Feuer A, Dinov ID (2018). Deep learning in pharmacogenomics: from gene regulation to patient stratification. Pharmacogenomics..

[157] Chiu Y-C, Chen H-IH, Gorthi A, Mostavi M, Zheng S, Huang Y, et al. Deep learning of pharmacogenomics resources: moving towards precision oncology. Brief Bioinform. 2020;21(6):2066–83. 10.1093/bib/bbz144 PMC7711267 31813953

[158] Rampášek L, Hidru D, Smirnov P, Haibe-Kains B, Goldenberg A. Dr.VAE: improving drug response prediction via modeling of drug perturbation effects. Schwartz R, editor. Bioinformatics. 2019;35:3743–3751. 10.1093/bioinformatics/btz158 PMC6761940 30850846

[159] Robinson MD, McCarthy DJ, Smyth GK (2010). edgeR: a Bioconductor package for differential expression analysis of digital gene expression data. Bioinformatics..

[160] Kompa B, Snoek J, Beam AL (2021). Second opinion needed: communicating uncertainty in medical machine learning. NPJ Digit Med.

[161] Wang H, Yeung D-Y (2016). Towards Bayesian deep learning: a framework and some existing methods. IEEE Trans Knowl Data Eng.

[162] Danaee P, Ghaeini R, Hendrix DA (2017). A deep learning approach for cancer detection and relevant gene identification. Biocomput 2017 [Internet].

[163] Khairnar P, Thiagarajan P, Ghosh S. A modified Bayesian convolutional neural network for breast histopathology image classification and uncertainty quantification. ArXiv201012575 Cs Eess [Internet]. 2020; [cited 2021 May 10]; Available from: http://arxiv.org/abs/2010.12575.

[164] Abdar M, Samami M, Mahmoodabad SD, Doan T, Mazoure B, Hashemifesharaki R, et al. Uncertainty quantification in skin cancer classification using three-way decision-based Bayesian deep learning. Comput Biol Med. 2021;135:104418. 10.1016/j.compbiomed.2021.104418 34052016

